# Removing Ocular Movement Artefacts by a Joint Smoothened Subspace Estimator

**DOI:** 10.1155/2007/75079

**Published:** 2007-12-06

**Authors:** Ronald Phlypo, Paul Boon, Yves D'Asseler, Ignace Lemahieu

**Affiliations:** ^1^The Medical Image and Signal Processing (MEDISIP) Group, ELIS Department, Faculty of Engineering Sciences (Firw), Ghent University, The Institute for Broadband Technology (IBBT), Sint-Pietersnieuwstraat 41, 9000 Ghent, Belgium; ^2^Department of Neurology, The Laboratory for Clinical and Experimental Neurophysiology (LCEN), Ghent University Hospital 10K1, De Pintelaan 185, 9000 Ghent, Belgium

## Abstract

To cope with the severe masking of background cerebral activity in the electroencephalogram (EEG) by ocular movement artefacts, we present a method which combines lower-order, short-term and higher-order, long-term statistics. The joint smoothened subspace estimator (JSSE) calculates the joint information in both statistical models, subject to the constraint that the resulting estimated source should be sufficiently smooth in the time domain (i.e., has a large autocorrelation or self predictive power). It is shown that the JSSE is able to estimate a component from simulated data that is superior with respect to methodological artefact suppression to those of FastICA, SOBI, pSVD, or JADE/COM1 algorithms used for blind source separation (BSS). Interference and distortion suppression are of comparable order when compared with the above-mentioned methods. Results on patient data demonstrate that the method is able to suppress blinking and saccade artefacts in a fully automated way.

## 1. INTRODUCTION

Recording of cerebral activity by means of the electroencephalogram (EEG) is a widespread technique that is well embedded in today's healthcare environment. The potentials recorded at the patient's scalp are a direct reflection of cerebral activity patterns and thus may serve as an indication to neurological diseases such as epilepsy, encephalopathies and sleep disorders. Being a noninvasive technique with a high temporal resolution, it is also frequently used in experimental settings in neurophysiology and psychology, where responses to external stimuli are measured.

Although the first article mentioning EEG registration already dates from 1924 (Hans Berger), there still remain a lot of side effects, inherent to the recording, that are to be dealt with. The major issue to be tackled when preprocessing the EEG is the contamination of the signal by artefacts. The latter hamper the interpretation by physicians of the cerebral activity, since they are often many times larger in amplitude than the neuronal activity of interest. The most well-known interfering sources are power line noise, muscle activity, and ocular movements. The classical frequency bands of interest in the EEG are situated between half a Hertz (delta band lower limit) and approximately 35–40 Hz (gamma band upper limit), although studies are found where in upper limits of gamma activity in evoked potentials extend beyond the classical 35–40 Hz limits up to 80 Hz [1]. Even scalp effects up to 200 Hz have been recorded as reported in [2], tagged as oscillation in the high gamma frequency band (60–200 Hz) while test persons imagined they were singing. In general, though, power line noise is easy to deal with since the main spectral bands of interest are usually limited between 0 Hz and 35 Hz. Because in these cases there is no spectral overlap with the EEG bands of interest, a simple low-pass or Notch filter with cancellation at 50 or 60 Hz suffices for the elimination of this artefact. Muscle artefact suppression is harder to resolve since the frequencies are situated in the upper part of the EEG spectrum. Moreover, the activity of the muscles stems from fast changing polarization of different muscle fibers, displaying undeterministic (low autocorrelation) behaviour. From this point of view, De Clercq et al. [3] proposed to make use of the canonical correlation analysis method to reduce the influence of muscle activity in EEG recordings. For ocular movement artefact suppression several solutions have already been put forward. Nevertheless, we will focus on the latter, showing where previous techniques fail and can thus be ameliorated and how to validate these studies based on objective measures derived from simulated data.

Whenever ocular movements are present in the EEG, the underlying cerebral activity cannot be interpreted by the physician, the experienced EEG technician, or the automated file processor. In the past, many solutions have been presented to suppress artefacts as much as possible. One of the earliest techniques was to request the patients or subjects to move their eyes as little as possible in order to obtain a nice and clean EEG recording. However, working with children and disabled people, which are still the main groups in clinical settings, seemed quasi infeasible. Besides, the current trend is shifting more and more to long-term monitoring, a set up in which it is infeasible to ask the subjects to avoid moving their eyes during recording. Also, when considering task related potential distributions through event related potentials, the creation of an additional task (staring) decreases the amplitude and the visibility of the cerebral response in the grand average related to the primary task, see, for example, [4–6]. Therefore, rejection of trials or segments contaminated with ocular artefacts has been put forward as an alternative solution. Although very commonly used in the past, this technique suffers from huge data losses since spontaneous blinks occur at a ratio of approximately 20 per minute [7]. For statistical tests based on long-term recorded data, this loss of data is highly unwanted, since the statistical results would suffer from the absence of the discarded data epochs. The same holds for examinations of seizure onsets in long-term recordings of epileptic patients, a period that may be heavily contaminated by muscular and ocular artefacts [8]. Moreover, for some ERP processing the blinking of the eyes are temporally highly correlated with the cerebral response of interest. Hence, this would result in an unwanted rejection of epochs of interest. [9].

For these reasons and because of increasing computational resources, the trend is shifting toward artefact correction methods. Although correction would be beneficial and preferable to rejection for the reasons mentioned above, it is claimed that rejection is still to be preferred over correction in EEG recordings of children [10]. Some of the more widely spread correction methods in research and clinical uses are the temporal and spectral regression techniques [11–13] and source separation or extraction methods [14, 15]. The regression methods, differentiated based on details in the implementation, all start from a set of reference signals and calculate the weighted contribution of each of those references at the recording sites or electrodes [12, 13, 16]. Although these methods have proven to be able to cope with different eye movement artefacts in the EEG, the major drawback is still the cross contamination between the reference electro-oculogram (EOG) channels and the EEG channels. It cannot be guaranteed that the EOG is free from contributions of cerebral origin, because propagation takes place in the same way as the ocular potentials influence the scalp potentials or EEG. This can be simply explained by the electrical property of reciprocity. Therefore, it is required that the reference used to perform regression with is well chosen and appropriately preprocessed. Recent studies [16, 17] showed that there exist means to tackle this problem by using adapted versions of the EOG channels. However, the validity of these results in clinical data is questionable, since the findings are based on a construction model that is equivalent to the correction process, hence biasing the outcome toward the presented method.

Apart from these regression techniques, a lot of research has been done on blind source separation (BSS) models. The EEG, being narrowband potential measurements that are the resultant from current sources in the brain, can be described by the linear approximation of the Maxwell equations for volume conduction. This also implies that the measurements are an instantaneous reflection of the underlying activity, and thus no delays should be considered. The general linear and instantaneous mixture model is given by

(1) $$x\left(t\right)=As\left(t\right)+\eta \left(t\right),$$

where $x(t)\in {\mathbb{R}}^{M}$ are the measured data from m electrodes sampled at time instance t , $s(t)\in {\mathbb{R}}^{N}$ are the n sources, at time instant t , $A\in {\mathbb{R}}^{M\times N}$ is the linear mixing matrix, and $\eta \left(t\right)$ is the additive noise. The i th column of A , $a_{i}$ , is a measure for the spreading of the activity of the i th source in s , $s_{i}$ , to the scalp electrodes, that is, the so-called source topography. Since $x\left(t\right)$ and $s\left(t\right)$ can be seen as random samples sampled independently from a multivariate distribution, we omit the time index t throughout the subsequent work, with explicit usage only there where needed to interpret the variables or the equations they are involved in. The additive noise in (1), when not negligible, will be considered as one of the sources in the subsequent work, and thus the term $\eta \left(t\right)$ can and will be dropped.

In the midtwentieth century, the most commonly used model to solve for the estimated sources (and their corresponding topographies), given only the measurement data x , was that of principal component analysis (PCA) [18], a technique based on the well-known singular value or eigenvalue decomposition (SVD, resp., EVD, also known as the Karhunen-Loève transform in information theory). These methods try to estimate underlying sources, based on maximisation of variance in a decorrelation framework for the sources and their topographies. However, many researchers have pointed out that the constraint of orthogonality on the source topographies does not stroke with the reality of the physiological sources underlying the EEG. Indeed, there is no ground on which we should assume that physiological source topographies are mutually orthogonal. Nevertheless, some interesting results based on these assumptions have been published recently, for example, [16, 19, 20].

In the last decade, independent component analysis (ICA) has become a popular technique to decompose the EEG signal into cerebral and noncerebral source estimates. The extra assumption of maximal source independence has found a lot of support in the EEG research community. In contrast to PCA/SVD, the sources no longer need to have mutually uncorrelated topographies, meaning the topographies are no longer constrained to be orthogonal. To solve for the linear and instantaneous mixture model subjected to the constraint of maximal independence, the source estimates ${\hat{s}}_{i}$ for the sources $s_{i}$ in (1) can be obtained through maximisation or minimisation of an appropriate object function, called a contrast. There exist a lot of contrasts in literature that one could optimise for in order to obtain maximally independent sources. Among the most popular are those of Hyvärinen and Oja [21] (Kurtosis based with a nonlinearity in the updating function), Lee et al. [22] (maximum likelihood based), Belouchrani et al. [23] (joint diagonalisation over a specific set of matrices) and Comon [24] or Cardoso and Souloumiac [25] (both cumulant tensor based). The first application of ICA to solving the EEG problem came from Makeig et al. [14] where they attempted to separate the raw EEG signal into physiological sources. An extension thereof was given in [15]. The research presented in the latter has been based on patient data as well as simulations and shows some of the abilities of ICA in EEG applications. In spite of the use of PCA/SVD in the preprocessing step of ICA implementations, the so-called whitening, PCA/SVD itself is often regarded as an inferior BSS algorithm compared to ICA.

In this paper, we show that by careful selection of a parameter set we can use the benefits of SVD, namely, a limited number of samples needed to estimate the covariance matrices with a sufficient precision, to our advantage in EOG source interference suppression. Although the results obtained by a such decomposition suffer a lot from methodological artefacts. Therefore we introduce an additional estimation step carried out by ICA (we used JADE [23]) and merge both results through a joint smoothened subspace estimator. The method of Canonical Correlation Analysis (CCA) [26] is an excellent candidate method for the latter, since the linear combination between both subspaces is automatically calculated and prevents us from introducing prefixed weighting scalars. Moreover, the subspace estimator reduces the subspace to the smoothest components only. For objective validation we propose a dipole-based model with eye activity modelling based on the model described in [7].

For ocular artefact suppression, other methods, such as wavelet transforms [27], the use of neural networks [28], and advanced filtering techniques [29], have been proposed. However, they form a minority and the reports on their success (or failure) are not much discussed in literature. We like to inform the reader that the given list of methods is certainly not exhaustive, but the given background should suffice to demonstrate the weaknesses in the current methods and to support the strategy we opted for.

## 2. MATERIALS AND METHODS

### 2.1. Materials

The patient data were collected at the Laboratory for Clinical and experimental neurophysiology (LCEN), at the Ghent University Hospital (Ghent, Belgium). Data were used from ten patients. The EEGs were recorded using a Telefactor Beehive system at a sample rate of 200 Hz. Twenty-one electrodes were placed on the patients' heads according to the 10–20 international system, together with six electrodes covering the lower temporal regions. One patient, showing numerous eye movement artefacts, was chosen to display the results on patient data, while the others were used for resampling as discussed below in Section 2.2.

In the rest of the paper, we consider the EEG as a 27-channel recording as defined above. Nevertheless, the presented method is valid for all average reference-based recordings with a reasonable number of channels M, including frontal channels.

### 2.2. EEG simulation model

**Figure 1:**
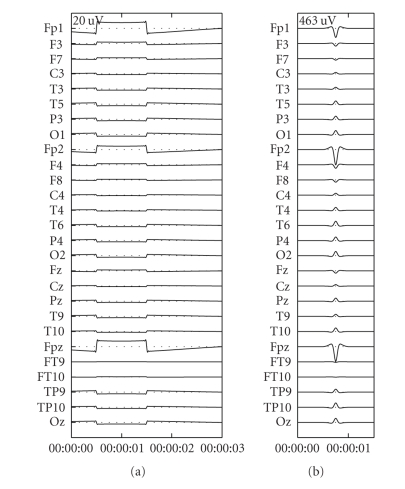
The potentials resulting from the simulated eye movements for horizontal saccade (a) and blinking (b).

The use of patient data has an enormous drawback in that there exists no way to qualify the performance of the algorithms, except for subjective scoring by physicians or experienced EEG readers. The latter are often ambiguous and suffer from interscorer variability [30]. We therefore propose to use a simulation model that simulates EEG data, given certain patient specifications. The method consists of building a dataset based upon forward modelling of dipoles. The calculation of the electrical field created by randomly activated cortical dipoles in a three-layer spherical head model (brain tissue, skull, and scalp) gives rise to the potentials at the respective electrodes. Each dipole is chosen to have a radial orientation, since the cortical activation patterns are known to be perpendicular to the cortical layers, a result of the physical layout of the pyramidal cortical cells [31]. The latter are then filtered according to spectral statistics derived from the patient data. An additional ocular dipole is added following the model in [7]. To add a waveform mimicking an eye blink we simulate the electrical shortcut that is created by the closure of the eyelid by a Gaussian bell curve and the eyeball rotation by a rotating dipole. An example of the resulting potentials due to these eye movements are given in Figure 1.

The strength of the model lies in the separate modelling of the activity of interest e , which is actually the artefact source, and the background activity b . By consequence, any method that has as a goal to separate the activity of interest from the background EEG can be evaluated using this model in combination with appropriate measures. The most simple measure would be the direct comparison between the background b and its estimate $\hat{b}$ returned by the method under investigation. The difference, which can also be expressed as a function of an estimate of the activity of interest $\hat{e}$ , may be given as the average sample distance asd :

(2) $$\text{asd}=\frac{1}{M}\sum_{i=1}^{M}\text{E}\left\{\sqrt{{\left(b-\hat{b}\right)}^{2}}\right\}=\frac{1}{M}\sum_{i=1}^{M}\text{E}\left\{\sqrt{{\left(e-\hat{e}\right)}^{2}}\right\},$$

where b , e , $\hat{b}$ , and e^∈ℝM are the samples of the background or the activity of interest used either by the simulation model or estimated by the algorithm under investigation, respectively. This measure, although attractive because of its simplicity, does not reveal any details about the source of error. Therefore we will turn to more sophisticated error measures to compare different methods and their performance, see 2.8. However, the asd measure will further on be used to estimate appropriate parameter settings for the pSVD method, as discussed in 3.1 and for evaluation of the algorithms under noise and relative scaling of b with respect to e .

To have a measure of performance in different scenarios, we define the SNR level as the mean ratio between the background signal and the eye movement at the samples where contamination occurs. Consider the set of all time instances t in the observed window W , we can take a subset containing only artefact contaminated samples t∈T(⊂W) . The definition of SNR is then given as follows:

(3) $$\text{SNR}=10\;\log_{10}\frac{{\left\|e_{t\in 𝒯}\right\|}_{F}^{2}}{{\left\|b_{t\in 𝒯}\right\|}_{F}^{2}},$$

where ${\left\|\cdot \right\|}_{F}$ denotes the Frobenius norm of the observation matrix.

### 2.3. BSS

The model that is used in the linear BSS framework is based on a direct mixture model:

(4) x=As,

where the measurements $x\in {\mathbb{R}}^{M}$ are linear combinations of the sources $s\in {\mathbb{R}}^{M}$ through a mixture $A\in {\mathbb{R}}^{M\times M}$ . We assume an equal number of sources with respect to the measurements. In the latter case, the inverse of A exists whenever A is full column rank. The aim of BSS algorithms is to find an estimate of s , $\hat{s}$ , by estimating the unmixing matrix W . In the ideal case the matrix W would be equal to $A^{-1}$ , and thus the source estimates $\hat{s}$ would equal the original sources s in the mixture. However, in most cases the estimated sources are only an approximation to the real sources and it is up to the user to find out whether the approximation is sufficient for his application. These techniques are termed blind since they only use the available data x as prior information, although some authors suggest to call databased source separation techniques semi blind since one always has to start from some additional basic assumption(s) [32], see below in Sections 2.3.1 and 2.3.2. However, to avoid confusion with the field of communications, where semi blind is used for methods where some parts of the source signals are known *a priori*, we dissuade the use of the terminology in any circumstances where the latter is not the case.

#### 2.3.1. Piecewise SVD

In a decomposition based on SVD, the additional basic assumption is the decorrelation or linear independence of the sources as well as of the topographies while pursuing maximal variance of the estimated sources. The temporal and spatial decorrelations are derived from the left and right correlation matrices of X , where X is the stacking of all samples $x\left(t\right)$ in a rectangular window of size T . If we denote by $t_{0}$ the first sample index of the window, then $X=\left[x\left(t_{0}\right)x\left(t_{0}+1\right)\cdots x\left(t0+T-1\right)\right]$ . The decomposition of the data is given as

(5) $$X=U\Sigma V^{T},$$

with $X\in {\mathbb{R}}^{M\times T}$ the measurements, U and V the eigenvectors of ${\mathrm{XX}}^{T}$ and $X^{T}X$ , respectively, and a matrix Σ containing the therewith associated singular values on its diagonal. The columns of U can be seen as the (mutually uncorrelated or orthogonal) source voltage distribution maps at the electrodes known as topographies. The columns of V are the (mutually uncorrelated or orthogonal) source activations and the i th singular value $\sigma_{i}$ on the diagonal of Σ is a measure for the explained variance by the corresponding i -th source in the original measurement data X . The sources are ordered according to their nondecreasing associated $\sigma_{i}$ with increasing index i .

When using the SVD in our source separation model we will use it in a sliding window of T=32 samples (160 milliseconds), moving with 8 samples per window position. The chosen windowing parameters are justified in 3.1. For each window d the SVD decomposition is calculated and the source topography $u_{1}$ associated with the source $v_{1}$ with maximal variance ( $\sigma_{1}$ ) is checked upon Criterion 1.

**Criterion 1**

The signal X is deflated by a topography $u_{1}\left(d\right)$ iff $\arg\;\underset{}{\max_{j}}\;c_{j}=\text{a}\text{b}\text{s}{(topo}_{j}^{T}u_{1}\left(d))/(|{topo}_{j}∥u_{1}(d)|\right)\geq 0.6.$

In the above criterion, deflation of X is performed by setting the corresponding $\sigma_{1}\left(d\right)$ to zero in the reconstruction (cf. (5). The template library containing the vectors ${\mathrm{topo}}_{j}$ is given in Table 1. This library is build from vectorially transcribed versions of the descriptions of spatial maps associated to ocular activity as can be found in [33]. The topographies in the table are reduced to the affected electrodes only, unmentioned electrodes are set to zero in the reference spatial maps. The first four topographies in the library contain maps generally associated with blinks, while the last four describe the eye gazing and horizontal movements.

Criterion 1 is no more than thresholding the subspace correlation [34] between the first component in $U(d)\left(u_{1}(d)\right)$ and the template library composed of $\left[{\mathrm{topo}}_{1}{\mathrm{topo}}_{2}\cdots {\mathrm{topo}}_{8}\right]$ as given in Table 1. The fuzziness included in the threshold ( 0.6 ) is due to the generality of the library and the mismatch between the true correlation and the estimated calculated correlation from an SVD based on 32 samples only. The short time windows are chosen as such as to cope with the nonstationarity of the EEG that is caused by the waxing and waning of sources in the background. The window of 32 samples or 160 milliseconds is a tradeoff between the oscillatory processes of 80–100 milliseconds [35] and a sufficient sample size for the SVD calculation, see Section 3.1 for more details. The reconstructed EEG with the locally deflated subspaces is then calculated for the first 8 samples of window d , given the results calculated for the windows d-3⋯d-1 , as the mean of these local reconstructions:

(6) $$X_{i\cdots i+7}^{D}=\frac{1}{4}\sum_{j=0}^{3}U\left(d-j\right)\Sigma^{D}\left(d-j\right)V_{i+8j+1\cdots i+8\left(j+1\right)}\left(d-j\right),$$

where $\Sigma^{D}$ denotes putting the first eigenvalue on the diagonal of Σ to zero when required so by the deflation criterion (Criterion 1). The i -th sample is the first sample of window d . The final result $X^{D}$ is then the concatenation of all the subwindows d that are corrected as above in (6). From here on we will call this deflation method piecewise SVD (pSVD) [20] referring to the window per window deflation approach.

#### 2.3.2. ICA

ICA algorithms try to find a decomposition based on the constraint of maximal statistical independence between the sources. In EEG, we may assume the model to be linear and instantaneous as given in (1) if the sources are stationary during the observation. Assume for a while that this constraint has been met. The maximal statistical independence of the sources is then a weaker constraint than the one used in the SVD, in the sense that there are no assumptions made about the topographies. It involves also an indeterminacy concerning permutation and scaling of the sources [24]. For this reason we cannot rely on the ordering of the components in the decomposition nor on the variances of the estimated sources for our selection criteria. Hence, we test all the component topographies of the decomposition against the decision rule in criterion 1. When testing all the topographies upon their subspace correlation with the template library we might find that multiple components obey the Criterion. In that case the data gets deflated by the subspace containing all these components. This can be done in the reconstruction of x (4) by setting all columns in A associated to the sources in s containing ocular activity (according to Criterion 1) to zero. The obtained cleaned dataset will subsequently be called $X_{\text{ICA}}^{D}$ .

ICA decompositions were taken from windows of 2000 samples or 10 seconds at 200 Hz sampling rate. This is a sufficient tradeoff between the nonstationarity and the samples needed to obtain an appropriate decomposition [36]. We here use the algorithm of JADE/COM1 [24, 37] because of its stability and its statistically robust approach. The algorithm does not suffer from initialisation, nor from parametrisation issues.

### 2.4. Joint smoothened subspace estimation

In a last step, both the estimates of the ocular components, $X_{\text{SVD}}^{D}$ and $X_{\text{ICA}}^{D}$ , are fed to a joint smoothened subspace estimator (JSSE). The algorithm that lends itself best to calculate the joint smoothened component(s) is the canonical correlation analysis (CCA). Let the subspaces estimated by the SVD and the ICA algorithm be Y and Z , respectively. Since there is no linear component from the pSVD algorithm that can be estimated (the general mixing matrix is nonexisting, it is a chain of short time linear mixtures), we use the piecewise back projected versions of the components onto the original EEG subspace, that is $y=x-x_{\text{SVD}}^{D}$ , to represent the subspace Y . If we take as a basis for Z , $z=x-x_{\text{ICA}}^{D}$ , we can calculate a common, smoothened component for the subspaces Y and Z by calculating the CCA for the joint subspace. For this, we proceed as follows (see also [26, 34] for more details on the canonical correlation analysis and calculation of angles between subspaces).

1. Take the QR decomposition of the joint signal space of the stacked matrix $P=\left(\begin{array}{c} Y\\ Z \end{array}\right)$ , where $Y=\left[y\left(t_{0})y(t_{0}+1\right)\cdots y(t_{0}+T)\right]$ and $Z=\left[z\left(t_{0})z(t_{0}+1\right)\cdots z(t_{0}+T)\right]$ . The joint signal subspace can be found by taking the SVD of $P=U_{s}\Sigma_{s}V_{s}^{T}$ and truncating at a noise level of 1%, that is $P^{D}=U_{s}\Sigma_{s}^{D}V_{s}^{T}$ , where $\Sigma_{s}^{D}$ describes the signal subspace formed by retaining only the p highest eigen values which acumulate to 99% of the total energy and where the relative energy is calculated as $\underset{i\in \;\text{Set}}{\sum }\sigma_{i}^{2}/\overset{M}{\underset{i=1}{\sum }}\sigma_{i}^{2}$ . The QR decomposition of these sources results in $V_{s}^{T}=Q_{0}R_{0}$ . Repeating this for a time delayed version of P gives $V_{s}^{T}(\tau )=Q_{\tau }R_{\tau }$ , where both $Q_{0},Q_{\tau }\in {\mathbb{R}}^{N\times N}$ are orthogonal and both $R_{0},R_{\tau }\in {\mathbb{R}}^{N\times M}$ are quasi upperdiagonal. τ is taken one sample period.
2. Calculate the SVD of $Q_{0}^{T}Q_{\tau }=U_{q}\Sigma_{q}V_{q}^{T}$ .
3. The joint and smoothened component is then found by taking $\sigma_{1\cdots j}U_{q,1\cdots j}^{T}R_{0}$ , where $\sigma_{1\cdots j}$ denote the first j entries in the diagonal of Σ put on the diagonal in a j×j matrix and $U_{q,1\cdots j}$ are the first j columns of $U_{q}$ .

The number of components j that are to be retained depends on the angles between the estimated components and the two subspaces. The cosine of these angles are given in descending order on the diagonal of Σ . To describe the common subspace between both Y and Z , it suffices to limit the number of components to the ones that are exceeding a subspace correlation of 0.9.

To find the source contribution in the original dataspace, we have to calculate back each of the estimated source contributions. The estimated source can be expressed as

(7) s^=Uq,1TQ0T=Uq,1T(VsDR0−1)T=Uq,1T(R0−1)TVsDT=Uq,1T(R0−1)T(ΣsD)−1UsTP.

From (7), it can be seen that the source can be expressed as a function of Y and Z , with a mixing matrix that is equal to $U_{q,1}^{T}{\left(R_{0}^{-1}\right)}^{T}{\left(\Sigma_{s}^{D}\right)}^{-1}U_{s}^{T}$ . The expression of Y as a function of the original data is piecewise linear which makes the resulting sources in s^ a weighted sum of a piecewise linear mixture (pSVD) and a completely linear mixture (JADE) of the original data in x .

### 2.5. Reconstruction phase

To have an adequate reconstruction of our artefact free data, we need to deflate our original dataspace by the projection of both subspaces Y and Z projected on the common subspace as returned by JSSE. Since these subspaces are a mixture of stationary, linear mixing and a nonstationary (and thus temporal nonlinear) mixing, respectively, the mixing matrix should be evaluated piecewise (i.e., temporally nonstationary), see (6) and (7). However, the estimated component ${\hat{s}}_{i}$ returned by the JSSE can be seen as being linearly mixed in the data, since it is itself already a combination of stationary and nonstationary source estimates ( y and z , and their respective stationary and nonstationary mixing matrix, as stated above). Hence, we might calculate its contribution as the least squares estimate between our original data x and a linear mixture $h_{i}\in {\mathbb{R}}^{M}$ of the component ${\hat{s}}_{i},i\in \left\{i,\forall i:\left|\Sigma_{ii}\right|\geq 0.9\right\}$ . This is given by

(8) $$h_{i}=\arg\;\underset{h_{i}}{\min}\;E\left\{{\left\|x-h_{i}{\hat{s}}_{i}\right\|}_{2}\right\}.$$

To deflate the dataspace X (associated with x ) by the subspace H spanned by the vectors $h_{i}$ , we use an iterative procedure, replacing x in (8) by the current estimate of the background activity b^(k) ( b^(1) being x itself). Subsequently the projection of ${\text{h}}_{i}$ onto the already calculated subspace $H^{p}=\text{span}\left[h_{1}h_{2}\cdots h_{i-1}\right]$ is subtracted from $h_{i}$ by using a Gram-Schmidt orthogonalisation procedure [34, pages 230–232]. The new estimate of the background estimate is then calculated as

(9) $$\hat{b}\left(k+1\right)=\left(I-h_{i}{\left(h_{i}^{T}h_{i}\right)}^{-1}h_{i}^{T}\right)\hat{b}\left(k\right),$$

where I is the identity matrix in ${\mathbb{R}}^{M\times M}$ and ${\left(h_{i}^{T}h_{i}\right)}^{-1}h_{i}^{T}$ is the (left) Moore-Penrose pseudoinverse of $h_{i}$ .

### 2.6. Alternatives to the proposed method

As noted in the introduction, the concept of ICA can be approached in different ways, yet leading to the same objective of mutual information reduction or maximal mutual independence. For comparison we include three ICA algorithms based on different point of views on statistical independence, that is, FastICA, JADE/COM1, and SOBI.

#### 2.6.1. FastICA

FastICA [21] is probably the most widely spread ICA method in various research communities. The popularity of FastICA can be explained mainly through its ease of use and the various possibilities to manipulate the objective, see [15, 38, 39] amongst others. Basically, the algorithm is supported by the general definition of statistical independence, saying that variables are independent if they are uncorrelated through every function. Furthermore, the method makes use of the optimal decorrelation function, namely the inverse cumulative density function of the source variables. Both assumptions are united in the decorrelation of the output of a fixed nonlinear function (e.g., tanh, $x^{4}$ ) of the prewhitened data. This has been shown to be similar to maximising the kurtosis of the estimated sources in case the nonlinear function approximates the inverse cumulative density function.

#### 2.6.2. JADE or COM1

The JADE [25] and COM1 [24] algorithms are both based on the maximisation of the marginal source cumulants by minimising the cross-cumulants of fourth order, either by jointly diagonalising tensor slices (JADE), either by pairwise processing of the entry signals (COM1). The idea originates from the Edgeworth expansion of the density functions, providing a sufficient statistic when truncated at order four. Both algorithms return equal performance rates and differ mainly in computational complexity [24, 37].

#### 2.6.3. SOBI

Relying solely on second order techniques, SOBI is an ICA algorithm using spatial as well as temporal information from the observed dataset^1^. The objective is to jointly decorrelate the data spatially and temporally, based on the information in the autocorrelation matrices of the data $R_{X\left(0\right)X\left(\tau \right)}$ . The input to the algorithm requires an additional set of time lags upon which SOBI will act. For this work, the set of time lags τ has been chosen as $T=\left\{\tau_{i}|0\leq \tau_{i}\leq 4*T_{s},\forall i\in \mathbb{Z}\right\}$ , where $T_{s}$ is the sampling period of the signal.

### 2.7. pSVD

Although not an ICA algorithm, we have a closer look at pSVD as it is one of the basic methods underlying JSSE. It is mainly used here to contrast the performance of pSVD outside, respectively, within the JSSE framework.

### 2.8. Evaluation measures

For a comparison, we evaluate the method and put its outcome next to that of the underlying basic techniques of pSVD and FastICA and the alternatives JADE and SOBI. Since simple measures such as asd do not suffice for a detailed error evaluation, we opt for performance measures as they are given in [40]. The measures take into account the source interferences, the methodological artefact and the total distortion. For clarity the definitions of the measures used here are repeated from [40] below.

**Definition 1**

Source to interference ratio is given as $SIR=10\,{\;\log}_{10}(∥s_{t}{∥}_{F}^{2}/∥e_{i}{∥}_{F}^{2}).$

**Definition 2**

Source to artefact ratio is given as $SAR=10\,{\;\log}_{10}((∥s_{t}+e_{i}{∥}_{F}^{2})/∥e_{a}{∥}_{F}^{2}).$

**Definition 3**

Source to distortion ratio is given as $SDR=10\,{\;\log}_{10}(∥s_{t}{∥}_{F}^{2}/(∥e_{i}+e_{a}{∥}_{F}^{2})).$

Where $\|\cdot {\|}_{F}$ denotes the Frobenius norm of its argument, ${\text{s}}_{t}$ is the source estimate, and ${\text{e}}_{i}$ and ${\text{e}}_{a}$ are the interference and artefact error, respectively.

The advantages of this set of measures is that it splits up the error in the estimated source s^j into a contribution that is related to the projection on the original source space of $s_{j}$ ( $s_{t}$ ), a projection of s^j on the subspace spanned by the vectors $s_{k},\forall k\neq j$ (interference $e_{i}$ ) and an artefactual source that is the projection on the remaining subspace which cannot be explained by any of the above two projections (artefact $e_{a}$ ). The latter is directly related to our methodologically introduced error or to numerical (round off) errors. The definitions of the above-mentioned measures resemble the familiar SNR definitions but are slightly altered to share mutually as little information as possible.

Note that we have omitted the noise term in all definitions, because we do not evaluate any noise perturbation studies. Noise perturbation studies of the pSVD algorithm can be found in [20] and for the JADE/COM1 algorithm in [24], amongst others.

## 3. Results

### 3.1. Parameter settings

To have an optimal parameter set for pSVD we minimalise the asd on a group of 250 simulated datasets over a set of correlation parameters and window lengths. Since we want to keep the library as general as possible, no changes are made in the spatial reference maps ${\mathrm{topo}}_{i},\forall i:1\leq i\leq 8$ , restricting the tuning to the two parameters mentioned above. The correlation parameter ϑ and the window length T were varied independently, whereupon the minimum asd (mean over the 250 datasets) was found at a window length of T=32 with a correlation threshold ϑ=0.7 .

**Figure 2:**
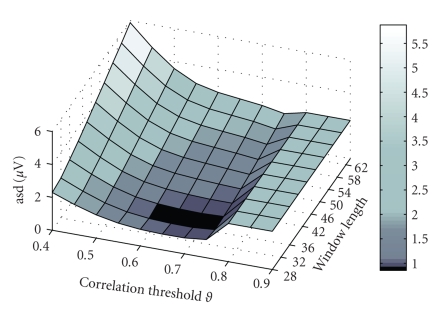
The asd as a function of the correlation threshold ϑ and of the window length T .

Figure 2 shows the mean values of asd as a function of T and ϑ . The minimum is reached at T=32 and ϑ=0.7 , respectively. Nevertheless, from hereon a threshold value of ϑ=0.6 is chosen in Criterion 1 to make the method as robust as possible to small changes in the data. It can be seen from Figure 2 that this small alteration in ϑ does not change a lot in the final asd value, but it will ensure a better performance in patient data where there is a higher effect of interfering background activity. Taking a value that is greater than 0.75 results in a value that is equal to no change, that is there is no component that will be identified as being close enough to the template library. From Figure 2 it is clear that the optimal window length is 32. Increasing the number of samples suffers from the orthogonality constraint and the stationarity assumptions that are made during this long-lasting window, decreasing the number of samples will result in an insufficient sample size for a robust estimation of the correlation.

### 3.2. Simulated data

**Figure 3:**
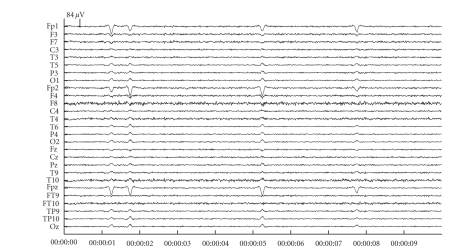
An example of a simulated dataset with blinks at an average of 5 dB above the background EEG level (SNR −5 dB).

**Figure 4:**
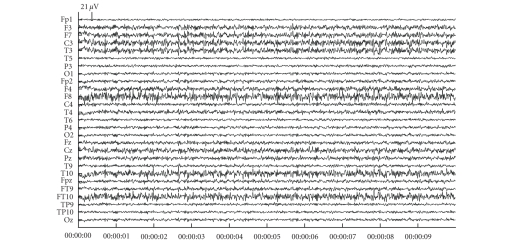
The cleaned simulation dataset.

**Figure 5:**
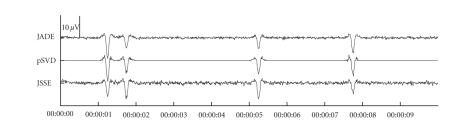
The first extracted source as estimated by JADE, pSVD, and JSSE. The ordening by JADE was done with descending kurtosis.

We show the consecutive steps for the artefact reduction with JSSE on a simulated dataset with an SNR of −18 dB. Figure 3 shows the simulated dataset consisting of the background EEG and ocular artefacts. There are 4 blinks in the dataset with varying amplitude and varying topography (left and right eye blink). Figure 4 shows the results of JSSE acting on the dataset in Figure 3. In Figure 5, the intermediate estimated sources are displayed for JADE and pSVD together with the final estimate through their combination using JSSE.

**Figure 6:**
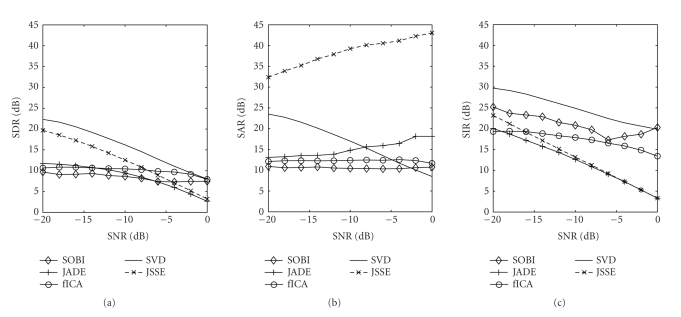
Values of SDR (a), SAR (b), and SIR (c) as a function of the SNR levels.

In Table 2, we show the results from 250 trials on simulated datasets for an SNR (see (3)) range of −20 dB to 0 dB. We compare the combined subspace method JSSE to the underlying algorithms that provide the subspace estimation (JADE and pSVD) and the two proposed alternatives SOBI and FastICA. To see the behaviour of the algorithms as a function of the SNR values of the datasets, we set out SDR, SAR and SIR values against SNR in Figure 6.

### 3.3. Patient data

**Figure 7:**
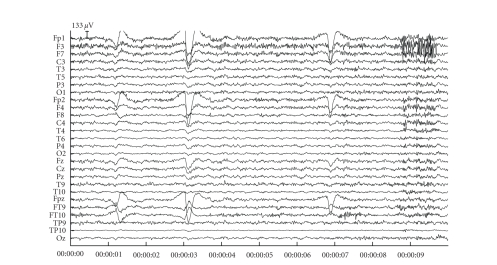
An example fragment of blinking artefacts. The blinks are clearly visible at seconds 1, 3, and 7.

**Figure 8:**
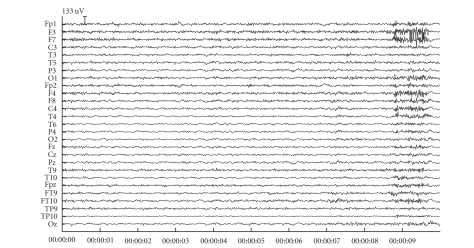
The results after having subjected the blink fragment of Figure 7 to JSSE.

**Figure 9:**
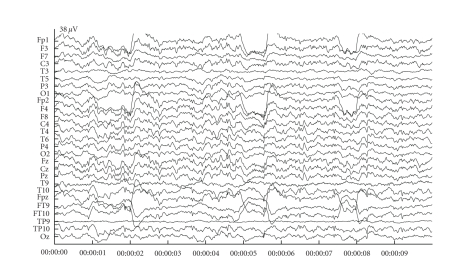
An example fragment of saccades. The saccades are clearly visible at seconds 1, 5, and 8.

**Figure 10:**
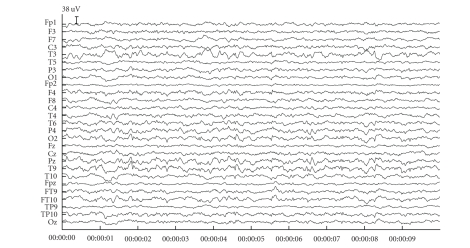
The results after having subjected the saccade fragment of Figure 9 to JSSE.

**Figure 11:**
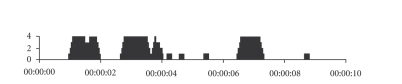
The pSVD profile associated with the blink fragment of Figure 7. The bars denote the number of deflations that occurred for each 8 sample window (with a maximum of 4 occurrences, see text).

**Figure 12:**
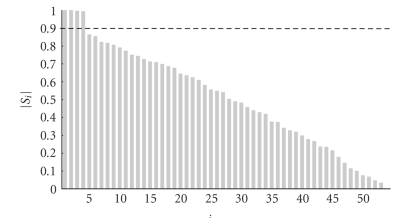
The spectrum of the subspace angles as obtained by the blink frame of Figure 7. The dashed horizontal line denotes the threshold level of 0.9.

Figures 7 and 9 contain two snippets of patient datasets recorded at the Ghent University Hospital. Figure 7 contains clear blinking artefacts at seconds 1, 3, and 7, whereas Figure 9 contains clear saccades at seconds 1, 5, and 8. Both dataframes have been subjected to JSSE of which the obtained results can be seen in Figures 8 and 10, respectively. For clarity, the spectrum of JSSE that accompanies the results in Figure 7 (i.e., the values on the diagonal of Σ obtained at the second step of JSSE, see Section 2.4) are given in Figure 12 and a profile of the pSVD correction is given in Figure 11. The latter shows how many windows were deflated to reconstruct the current 8 samples.

**Figure 13:**
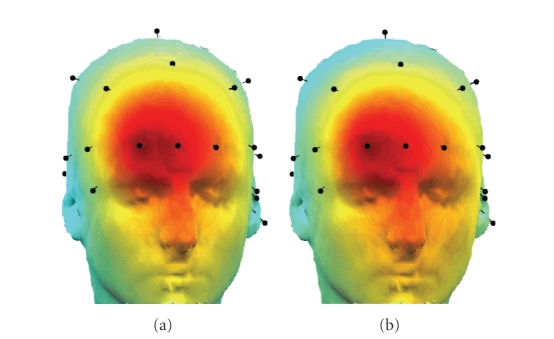
The topography of the first source of JADE (a) and an estimation of the JSSE topography, based on the reconstruction in (9) (b).

Figure 13 shows two scalp maps, representing the weighing of a source estimate of JADE, respectively, JSSE onto the scalp electrodes (both components were taken to correspond to the same eye movement, i.e., a right eye blink). The scalp map associated to the JSSE component is given by the entry $h_{i}$ in the topographical matrix H . Remember that a scalp map reflecting the activity of the eye movement estimated by pSVD cannot be given, since it includes nonstationarities which are inherent to the method of pSVD.

## 4. Discussion

Combining two statistical estimation algorithms through the JSSE results in an ameliorated eye movement estimation from the EEG. The motivation to use short time statistics (pSVD) to cope with the nonstationarity of the cerebral activity is justified in the sense that it results in a minimisation of interference from other sources present in the EEG (reflected in maximal SIR), although it might introduce too much artefactual components caused by its windowing (SAR). Using the prior that in most cases eye movements are independent from the cerebral processes, the results of JADE show a quite good artefact suppression—although lower than that of pSVD—in the considered window (SAR) but are disappointing with respect to the interference suppression (SIR). The introduction of the joint and smoothened subspace estimation offers a solution hereto by augmenting the SAR through joining the advantages of both techniques. The results in Table 2 and Figure 6 show that the extracted component results in an interference and distortion suppression that are close to the pSVD results, while the enoying windowing artefact is suppressed outstandingly in its combination with JADE through the JSSE.

From Figure 6, it can be seen that the price to pay for a such amelioration in artefact suppression is an approximately constant 3 dB loss in SDR with respect to piecewise corrected EEG (pSVD). For the interference suppression, this even runs up to 17 dB (at an SNR level of 0 dB), although being acceptable at reasonable SNR levels (approx., 8 dB at −15 dB SNR). We thus have to give in on both SDR and SIR if a gain in SAR is of importance. From Figure 11 it can be seen that methodological artefacts can be introduced quite easily by the windowing that is inherent to the pSVD method. Since the first component of each local SVD decomposition does not necessarily the same spatial projection, the reconstruction introduces discontinuities related to the windowing. The reconstruction being influenced by a possible nonstationarity of the eye movement vector, nonstationarities in the background activity as well as the on/off switching caused by the binary decision process. The latter is directly reflected in the artefact error and by consequence in both the SDR and SAR values.

Experiments on patient EEG showed promising results concerning the suppression of blinks and saccades. Although it is difficult, or even impossible, to show objective measures for evaluation, we observe that the estimated topographies are close to the topographies as estimated through JADE. An exemplar topography as in Figure 13 shows that the topography is spatially even more concentrated around the eye, pointing at a closer to dipole behaviour, which is in line with the model proposed in [7].

From Figure 12 it can be seen that the chosen threshold for the spectrum of the JSSE falls in the spectral gap. Although this is not always the case, the threshold at 0.9 offers a reliable reconstruction in the majority of the cases as proved by the simulation results and the two patient frames presented, where even on visual inspection it can be seen that the method leaves almost no traces at the time spots where it interacted on the EEG recording (see Figures 4, 8, and 10).

One could of course think of other subspace combining methods. The simplest form being the reconstruction of the EEG by taking a weighted sum of both partial reconstructions as they are given by JADE and pSVD, respectively. However, the disadvantage would be that the errors are added while JSSE has a more sophisticated error suppression with respect to the errors introduced by the two supporting methods (see Figure 6 and Table 2). Yet another combination method would be to take a threshold onto the profile provided by pSVD (as in Figure 11) and only consider the time instances of the ICA (in our case, this would be JADE) reconstruction that are labelled by this thresholding, leaving the remaining time instances untouched. Unfortunately, this does not resolve for the artefact suppression. On the contrary, the discontinuities will be more articulated if the threshold would be augmented (resulting in lower values of SAR), and the interference at the time instances considered will not exceed the performance of a regular ICA algorithm. Moreover, it is implicitly assumed that the background activity would be stationary along the complete frame (i.e., 10 seconds in our case), which is quite in contrast with the findings in [35].

## 5. Conclusion

This study shows the importance of combining BSS techniques with different order statistics. It convincingly shows that merging short time signal characteristics (pSVD) with more global measures (JADE) into a joint smoothened subspace estimator (JSSE) provides acceptable to outstanding results compared to many of the commonly used standard ICA techniques. More specifically, it follows directly from our simulations that the proposed method is superior in artefact suppression, while it keeps up with the methods of FastICA, JADE, pSVD, and SOBI concerning the interference and distortion suppression, especially at low (highly negative) SNR values.

The proposed method has proven to be capable of suppressing ocular artefacts in the EEG in a fully automated way, relying on a set of patient-independent reference topographies as a prior.

## Figures and Tables

**Table 1:** The template library composed of column vectors (here transposed and limited to their core information) as used in Criterion 1.

	Fp1	Fp2	Fpz	F7	F8	FT9	FT10	F3	F4	C3	C4
${\mathrm{topo}}_{1}$	1	1	0.5	0	0	0	0	0	0	0	0
${\mathrm{topo}}_{2}$	1	0	0	0	0	0	0	0	0	0	0
${\mathrm{topo}}_{3}$	0	1	0	0	0	0	0	0	0	0	0
${\mathrm{topo}}_{4}$	0	0	1	0	0	0	0	0	0	0	0
${\mathrm{topo}}_{5}$	0	0	0	1	0	0	0	0	0	0	0
${\mathrm{topo}}_{6}$	0	0	0	0	1	0	0	0	0	0	0
${\mathrm{topo}}_{7}$	0	0	0	1	-1	1	-1	0	0	0	0
${\mathrm{topo}}_{8}$	1	1	1	0	0	0	0	1	1	1	1

**Table 2:** Results from 250 runs of simulations for which the mean was taken over SNR levels of −20 dB to 10 dB.

	SOBI	JADE	FastICA	pSVD	JSSE
SIR (dB)	21.71	14.93	17.74	26.06	17.07
SAR (dB)	10.58	15.51	12.28	18.98	39.51
SDR (dB)	8.50	9.36	10.16	17.97	14.80

## Footnotes

This is in contrast with all of the above algorithms which consider the observations as stochastic independent realisations and thus neglecting any possible temporal dependencies.

## References

[1] Lutz A, Greischar LL, Rawlings NB, Ricard M, Davidson RJ (2004). Long-term meditators self-induce high-amplitude gamma synchrony during mental practice. *Proceedings of the National Academy of Sciences of the United States of America*.

[2] Gunji A, Ishii R, Chau W, Kakigi R, Pantev C (2007). Rhythmic brain activities related to singing in humans. *NeuroImage*.

[3] de Clercq W, Vergult A, Vanrumste B, van Paesschen W, van Huffel S (2006). Canonical correlation analysis applied to remove muscle artifacts from the electroencephalogram. *IEEE Transactions on Biomedical Engineering*.

[4] Weerts TC, Lang PJ (1973). The effects of eye fixation and stimulus and response location on the contingent negative variation (CNV). *Biological Psychology*.

[5] Verleger R (1991). The instruction to refrain from blinking affects auditory P3 and N1 amplitudes. *Electroencephalography and Clinical Neurophysiology*.

[6] Ochoa CJ, Polich J (2000). P300 and blink instructions. *Clinical Neurophysiology*.

[7] Iwasaki M, Kellinghaus C, Alexopoulos AV (2005). Effects of eyelid closure, blinks, and eye movements on the electroencephalogram. *Clinical Neurophysiology*.

[8] Hallez H, Vergult A, Phlypo R (2006). Muscle and eye movement artifact removal prior to EEG source localization.

[9] Hillyard SA, Galambos R (1970). Eye movement artifact in the CNV. *Electroencephalography and Clinical Neurophysiology*.

[10] Somsen RJ, van Beek B (1998). Ocular artifacts in children's EEG: selection is better than correction. *Biological Psychology*.

[11] Fatourechi M, Bashashati A, Ward RK, Birch GE (2007). EMG and EOG artifacts in brain computer interface systems: a survey. *Clinical Neurophysiology*.

[12] Croft RJ, Barry RJ (2000). Removal of ocular artifact from the EEG: a review. *Neurophysiologie Clinique*.

[13] Schlögl A, Keinrath C, Zimmermann D, Scherer R, Leeb R, Pfurtscheller G (2007). A fully automated correction method of EOG artifacts in EEG recordings. *Clinical Neurophysiology*.

[14] Makeig S, Bell AJ, Jung T-P, Sejnowski TJ (1996). Independent component analysis of electroencephalographic data. *Advances in Neural Information Processing Systems*.

[15] Vigário RN (1997). Extraction of ocular artefacts from EEG using independent component analysis. *Electroencephalography and Clinical Neurophysiology*.

[16] Wallstrom GL, Kass RE, Miller A, Cohn JF, Fox NA (2004). Automatic correction of ocular artifacts in the EEG: a comparison of regression-based and component-based methods. *International Journal of Psychophysiology*.

[17] Wallstrom G, Kass R, Miller A, Cohn J, Fox N (2002). Correction of ocular artifacts in the EEG using Bayesian adaptive regression splines. *Bayesian Statistics*.

[18] Lins OG, Picton TW, Berg P, Scherg M (1993). Ocular artifacts in recording EEGs and event-related potentials II: source dipoles and source components. *Brain Topography*.

[19] Casarotto S, Bianchi AM, Cerutti S, Chiarenza GA (2004). Principal component analysis for reduction of ocular artefacts in event-related potentials of normal and dyslexic children. *Clinical Neurophysiology*.

[20] Phlypo R, van Hese P, Hallez H (2006). PSVD: a method for robust, real time eye movement artifact rejection from the EEG.

[21] Hyvärinen A, Oja E (1997). A fast fixed-point algorithm for independent component analysis. *Neural Computation*.

[22] Lee T-W, Girolami M, Sejnowski TJ (1999). Independent component analysis using an extended infomax algorithm for mixed subgaussian and supergaussian sources. *Neural Computation*.

[23] Belouchrani A, Abed-Meraim K, Cardoso J-F, Moulines E (1997). A blind source separation technique using second-order statistics. *IEEE Transactions on Signal Processing*.

[24] Comon P (1994). Independent component analysis. a new concept?. *Signal Processing*.

[25] Cardoso J-F, Souloumiac A (1993). Blind beamforming for non-Gaussian signals. *IEE Proceedings F*.

[26] Borga M, Knutsson H (2001). A canonical correlation approach to blind source separation. *Tech. Rep. LiU-IMT-EX-0062*.

[27] Krishnaveni V, Jayaraman S, Anitha L, Ramadoss K (2006). Removal of ocular artifacts from EEG using adaptive thresholding of wavelet coefficients. *Journal of Neural Engineering*.

[28] Erfanian A, Mahmoudi B (2005). Real-time ocular artifact suppression using recurrent neural network for electro-encephalogram based brain-computer interface. *Medical and Biological Engineering and Computing*.

[29] Puthusserypady S, Ratnarajah T (2006). Robust adaptive techniques for minimization of EOG artefacts from EEG signals. *Signal Processing*.

[30] Agarwal R, Takeuchi T, Laroche S, Gotman J (2005). Detection of rapid-eye movements in sleep studies. *IEEE Transactions on Biomedical Engineering*.

[31] Hallez H, van Hese P, Vanrumste B (2005). Dipole localization errors due to not incorporating compartments with anisotropic conductivities: simulation study in a spherical head model. *International Journal of Bioelectromagnetism*.

[32] Babaie-Zadeh M, Jutten C (2006). Semi-blind approaches for source separation and independent component analysis.

[33] Stern JM, Engel J (2004). *Atlas of EEG Patterns*.

[34] Golub GH, van Loan CF (1996). *Matrix Computations*.

[35] Freeman WJ (2004). Origin, structure, and role of background EEG activity—part 2: analytic phase. *Clinical Neurophysiology*.

[36] Hyvärinen A, Särelä J, Vigário R (1999). Bumps and spikes: artifacts generated by independent component analysis with insufficient sample size.

[37] Cardoso J-F (1999). High-order contrasts for independent component analysis. *Neural Computation*.

[38] Hesse CW, James CJ (2006). On semi-blind source separation using spatial constraints with applications in EEG analysis. *IEEE Transactions on Biomedical Engineering*.

[39] Lu W, Rajapakse JC (2001). ICA with reference.

[40] Vincent E, Gribonval R, Févotte C (2006). Performance measurement in blind audio source separation. *IEEE Transactions on Audio, Speech and Language Processing*.

